# Intrinsic Differences in Immune Checkpoint Inhibitor-Induced Myocarditis: A Retrospective Analysis of Real World Data

**DOI:** 10.3389/fphar.2022.914928

**Published:** 2022-07-05

**Authors:** Yanna Lei, Xiufeng Zheng, Qian Huang, Xiaoying Li, Meng Qiu, Ming Liu

**Affiliations:** Department of Abdominal Oncology, West China Hospital, Sichuan University, Chengdu, China

**Keywords:** immune checkpoint inhibitors, immune-related myocarditis, immune-related myositis, troponin, lactate dehydrogenase

## Abstract

Immune-related myocarditis is a severe and even life-threatening immune-related adverse event (irAE) which may also be underestimated due to the challenge in diagnosis. The inherent difference between individuals with immune-associated myocarditis has received little attention. Our study aimed to identify which baseline characteristics could contribute to distinguishing mild from severe ICI myocarditis. A retrospective analysis was conducted between March 2019 and June 2020 in West China Hospital, and 18 patients with immune-related myocarditis were studied. Patients were classified as having mild (*n* = 12) or severe myocarditis (*n* = 6), according to the clinical manifestations and hemodynamic complications. Factors associated with severe myocarditis were identified by comparing covariates derived from medical records in various groups. In this retrospective analysis, the median age of the 18 patients was 60 years old. Most myocarditis cases occur early and approximately after the first or second ICI infusion. The severity of myocarditis may be correlated with lactate dehydrogenase (LDH) (*p* = 0.04) and troponin levels (*p* = 0.0057). The relationship between troponin and myocarditis was further confirmed in another cohort, which included 30 patients. In addition, patients are more likely to develop multi-irAEs, and myositis was the most common second irAE. Those who experience multi-irAEs usually had significantly higher LDH (*p* = 0.02) and myoglobin levels (*p* = 0.02) than those who did not experience them. All patients were treated with steroids timely, and the mortality rate was 5.6% in our study. In this study, we explored risk factors for severe myocarditis and emphasized the importance of a multidisciplinary team in assisting diagnosis and treatment options. It is critical to initiate corticosteroid therapy, regardless of the severity of the myocarditis.

## Introduction

The advent of immune-checkpoint inhibitors (ICIs) which block cytotoxic T lymphocyte-associated protein-4 (CTLA-4) and the programmed death-1 (PD-1)/programmed death ligand-1 (PD-L1) axis, both as monotherapy or in combination strategies, have revolutionized cancer treatment (Pardoll, 2012; Postow et al., 2015; Gong et al., 2018; Liu and Guo, 2018). However, as the use of ICIs in clinical practice has increased, immune-mediated toxicities have been observed, which can impact numerous organ systems, including the heart (Escudier et al., 2017; Zhu et al., 2021). Immune-related myocarditis is the most serious life-threatening toxicity, and the underlying pathomechanism is poorly understood (Liu et al., 2021). The incidence of immune-related myocarditis caused by ICI is approximately 1%, but the mortality rate can be as high as 46% (Mahmood et al., 2018; Moslehi et al., 2018). Because of its rarity, information on this unusual disease is limited, and further research is needed to fully understand the devastating irAEs. Furthermore, patients’ data are scarce on the clinical characteristics, results of pertinent examinations, and outcomes of ICI-related cardiotoxicity.

To gain better knowledge of this disease, we analyzed and described the characteristics of patients with immune-related myocarditis, including baseline clinical characteristics, drug-related data, relevant examination results, and outcomes. Our research looked into the internal disparities between patients and identified baseline variables that might assist in differentiating between low- and high-severity ICI myocarditis.

## Materials and Methods

### Study Population

In this single-center retrospective study, data were collected from 18 patients with ICI-related myocarditis in West China Hospital from March 2019 to June 2020. According to the clinical manifestations and whether they experienced hemodynamic consequences such as heart failure, cardiogenic shock, or arrhythmia, the patients were divided into mild (*n* = 12) and severe (*n* = 6) myocarditis groups. Inclusion criteria were listed as follows: 1) a histologically confirmed diagnosis of solid malignancy in West China Hospital; 2) age ≥18 years at ICI initiation; 3) received at least one cycle of ICI; 4) met the diagnostic criteria through multidisciplinary panel decision; and 5) exclude the possible diagnosis of myocarditis caused by other reasons such as COVID. The study was approved by the institutional review board, and the requirement for written informed consent was waived. The present study was approved by the institutional review board of West China Hospital and was exempted from informed consent requirements owing to its retrospective design.

This article does not contain any studies with human participants or animals performed by any of the authors. All methods were performed in accordance with the relevant guidelines and regulations.

### Patient Data Collection

The following characteristics of patients were recorded: 1) clinical baseline characteristics: age, gender, smoking and alcohol status, comorbid conditions, disease stage, cancer type, and histological subtype; 2) ICI-related information: type of ICI, treatment start date, the date of diagnosis, therapy line, combination with other drugs, and response patterns; 3) relevant examination results: laboratory data, including myocardial markers and infection-related indicators, echocardiography, coronary angiography, and coronary computed tomography (CT); and 4) treatment and outcome.

### Statistical Analysis

Study variables were presented as number (n)—percentage (%) and mean ± standard deviation. Wilcoxon rank-sum test was used for continuous variables, and χ2 or Fisher’s exact test was used for categorical variables. Missing data were not analyzed in this study. The statistical analyses were conducted *via* R 4.0.3 and GraphPad Prism 8. *p* < 0.05 was a statistically significant standard.

## Results

### Baseline Characteristics

**FIGURE 1:**
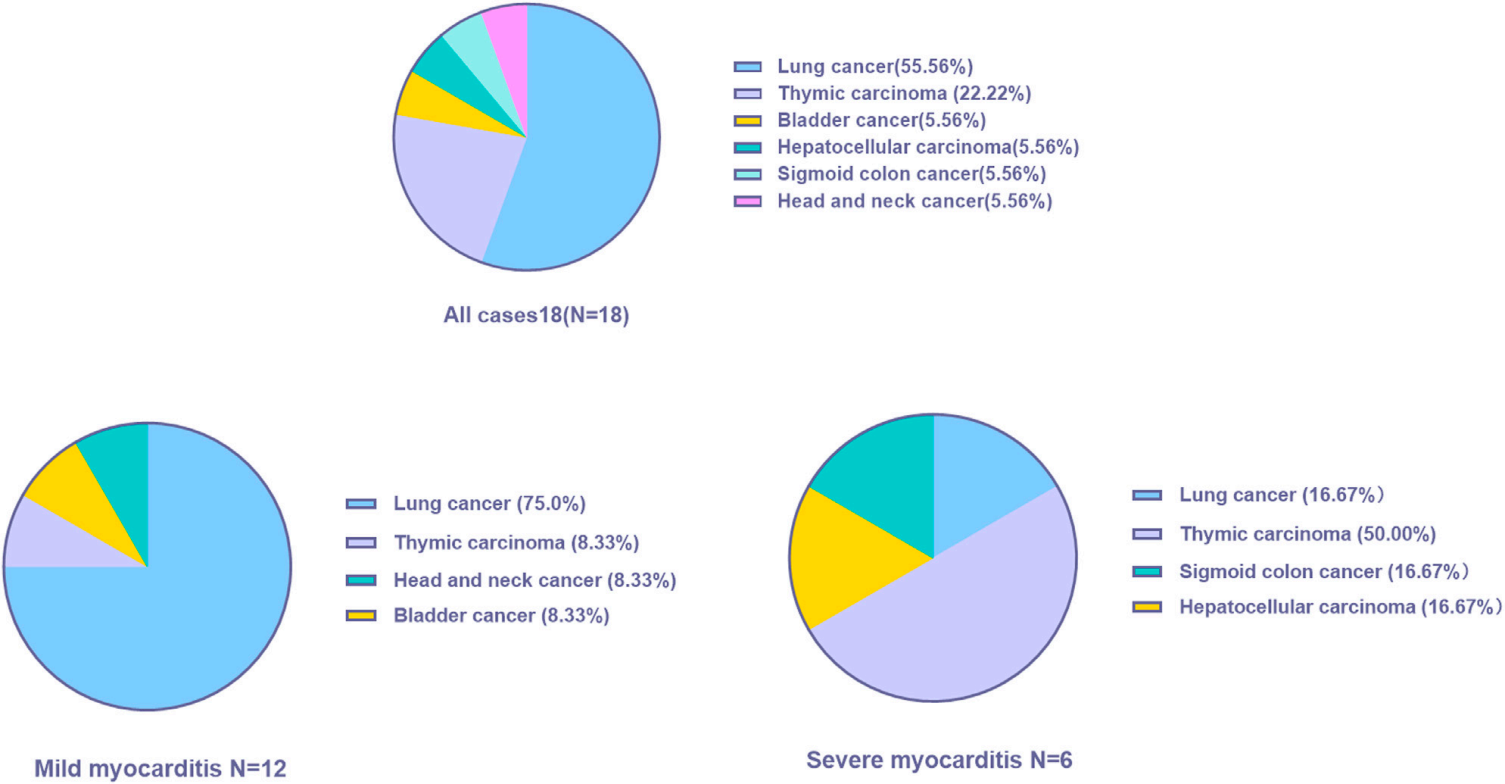
Cancer type distribution of all patients.

A total of 18 patients who met the selection criteria were retrospectively identified in this study, and the patients were classified into mild (*n* = 12) and severe myocarditis (*n* = 6) according to the clinical manifestation and whether they had hemodynamic complications, such as heart failure, cardiogenic shock, or arrhythmia. The clinical characteristics of the 18 patients with immune-related myocarditis are shown in Table 1. The median age among the 18 patients was 60 years old (range from 29.0 to 79.0 years), 83.3% were male, and a history of smoking was reported in 33.3%. Alcohol consumption was reported in 16.7% of the patients. 27.8% of patients had a history of diabetes, and 38.9% of patients had hypertension. Lung cancer was the most common cancer diagnosis (55.6%), and thymic carcinoma ranked next (22.2%), followed by bladder cancer (5.56%), sigmoid colon cancer (5.56%), and head and neck cancer (5.56%) (Figure 1). In addition, the majority of patients (72.2%) were in stage IV cancer at the time of ICI commencement. Half of the patients were asymptomatic. The most common clinical manifestations included dyspnea and fatigue.

**TABLE 1:** Clinical characteristics of the patients and information on ICI in patients with immune-related myocarditis.

	Mild group (*n* = 12)	Severe group (*n* = 6)	All cases (*n* = 18)	*p-*value
Age (years)				
Median [min, max]	61.5 [45.0, 79.0]	44.5 [29.0, 67.0]	60.0 [29.0, 79.0]	0.08
Gender				
Male	10 (83.3%)	5 (83.3%)	15 (83.3%)	1.00
Female	2 (16.7%)	1 (16.7%)	3 (16.7%)	
Smoking history				
Yes	5 (41.7%)	1 (16.7%)	6 (33.3%)	0.60
No	7 (58.3%)	5 (83.3%)	12 (66.7%)	
Alcohol history				
Yes	2 (16.7%)	1 (16.7%)	3 (16.7%)	1.00
No	10 (83.3%)	5 (83.3%)	15 (83.3%)	
Diabetes				
Yes	3 (25.0%)	2 (33.3%)	5 (27.8%)	1.00
No	9 (75.0%)	4 (66.7%)	13 (72.2%)	
Hypertension				
Yes	7 (58.3%)	0 (0%)	7 (38.9%)	0.04
No	5 (41.7%)	6 (100%)	11 (61.1%)	
Cancer type				
Lung cancer	9 (75.0%)	1 (16.7%)	10 (55.6%)	0.048
Thymic carcinoma	1 (8.3%)	3 (50.0%)	4 (22.2%)	
Other cancers	2 (16.7%)	2 (33.3%)	4 (22.2%)	
Cancer stage				
Stage IV	8 (66.7%)	5 (83.3%)	13 (72.2%)	0.24
Stage III	4 (33.3%)	1 (16.7%)	5 (27.8%)	
Clinical symptoms				
Yes	3 (25.0%)	6 (100%)	9 (50%)	0.141
No	9 (75.0%)	0 (0%)	9 (50%)	
Initial treatment line				
1st	9 (75.0%)	2 (33.3%)	11 (61.1%)	0.01
2nd	2 (16.7%)	0 (0%)	2 (11.1%)	
≥3rd	0 (0%)	4 (66.7)	4 (22.2%)	
Missing	1 (8.3%)	0 (0%)	1 (5.6%)	
ICI type				
Anti–PD-1	10 (83.3%)	5 (83.3%)	15 (83.3%)	1.00
Anti–PD-L1	2 (16.7%)	1 (16.7%)	3 (16.7%)	
Treatment category				
ICI alone	6 (50%)	3 (50.0%)	9 (50.0%)	0.42
Chemo + ICI	6 (50%)	2 (33.3%)	8 (44.4%)	
Targeted + ICI	0 (0%)	1 (16.7%)	1 (5.6%)	
Times to onset				
Mean (SD)	64.8 (52.3)	36.2 (20.6)	55.2 (45.7)	0.40
Median [min, max]	57.0 [4.00, 155]	32.5 [14.0, 69.0]	51.0 [4.00, 155]	
Number of ICI cycles				
1	4 (33.3%)	3 (50.0%)	7 (38.9%)	0.38
2	4 (33.3%)	2 (33.3%)	6 (33.3%)	
≥3	4 (33.3%)	1 (16.7%)	5 (27.8%)	
Mean (SD)	2.75 (2.22)	1.67 (0.82)	2.39 (1.91)	
Median [min, max]	2.00 [1.00, 8.00]	1.50 [1.00, 3.00]	2.00 [1.00, 8.00]	
Efficacy of ICIs				
PR	5 (41.7%)	0 (0%)	5 (27.8%)	0.299
SD	6 (50.0%)	4 (66.7%)	10 (55.6%)	
PD	1 (8.3%)	0 (0%)	1 (5.6%)	
Missing	0 (0%)	2 (33.3%)	2 (11.1%)	

ICI, immune checkpoint inhibitor; PD, progressive disease; PD-L1, programmed death-ligand 1; PR, partial response; SD, stable disease; PD, progressive disease. Tumor responses were assessed by computer tomography and determined by RECIST version 1.1.

In the mild myocarditis group, the mean age was 63.3 years old (range from 45.0 to 79.0 years), and in the severe group, the mean age was 46.8 years old (range from 29.0 to 67.0 years). Lung cancer was the most prevalent cancer type in the mild group (75.0%), but thymic carcinoma was the most common cancer type in the younger age group (50.0%). Furthermore, none of the patients with severe myocarditis had a history of hypertension.

Clinical signs of severe ICI-related myocarditis include arrhythmia, heart failure, and cardiogenic shock. Only one of the six patients with severe myocarditis in this study did not have an arrhythmia; the other five out of six had all of the clinical symptoms listed earlier.

### Treatment-Related Characteristics

The majority of patients (61.1%) received ICIs as first-line therapy, while chemotherapy, not immunotherapy, has been used as first- or second-line therapy in the severe myocarditis group. In 66.7% of patients with severe myocarditis, ICIs were used as a third-line or later treatment (Table 1). According to the distinct mechanisms of action of ICIs, 15 patients (83.3%) received anti-PD-1 therapy, and the remaining three patients (16.7%) received anti-PD-L1 treatment. The percentage distribution of 18 cases was as follows: pembrolizumab 33.3%, sintilimab 22.2%, camrelizumab 16.7%, and other ICIs 27.8%, according to various ICI administered.

The median cycles of the onset of immune-related myocarditis from the initiation of ICI infusion were 2 (range, 1–8 cycles), and the median time was 51.0 days (range, 4–155 days). The number of days from the initiation of ICI infusion to the presentation of myocarditis in the mild group was longer than in the severe group. The mean time was 64.8 and 36.2 days in the mild and severe groups, respectively. Approximately 72.2% of patients developed myocarditis after 1 or 2 ICI infusion cycles. For patients with severe myocarditis, only one patient received more than 3 cycles at the time of diagnosis. Tumor responses were assessed using computer tomography and determined using the RECIST version 1.1. Overall, five patients experienced partial responses, ten patients exhibited stable disease, and one patient had progressive disease after ICI administration.

Patients were divided into three groups based on their treatment regimen. The proportion of participants who used ICI alone, chemotherapy + ICI, and targeted therapy + ICI were 50.0%, 44.4%, and 5.60%, respectively. Due to the small number of participants in this study, people treated with ICI alone (*n* = 9) and Chemo + ICI (*n* = 8) will be discussed further (Table 2). The median onset time was earlier in the Chemo + ICI group compared with ICI alone cohort (34.5 vs. 69.0 days). In the mild-myocarditis group, the onset time of ICI alone was 109 days, which was longer than patients who received Chemo + ICI (onset time:34.5 days). Timing of the development of immune-related myocarditis with a mean delay of 78.9 days (range, 7–155 days) and a median of 2 doses from the first ICI administration in the monotherapy group. Details of other information in each subgroup are listed in Table 2.

**TABLE 2:** Comparison of treatment regimens in myocarditis patients.

	ICI alone (*n* = 9)	ICI + Chemo (*n* = 8)	*p-*value
Age			
Median [min, max]	60.0 [33.0, 79.0]	61.5 [29.0, 75.0]	0.96
Cancer type			
Lung cancer	3 (33.3%)	7 (87.5%)	0.08
Thymic carcinoma	3 (33.3%)	1 (12.5%)	
Other cancers	3 (33.3%)	0 (0%)	
Initial ICI treatment line			
1st	6 (66.7%)	5 (62.5%)	1.09
2nd	1 (11.1%)	1 (12.5%)	
≥3rd	1 (11.1%)	2 (25.0%)	
Missing	1 (11.1%)	0 (0%)	
Efficacy of ICIs			
PR	2 (22.2%)	3 (37.5%)	0.61
SD	6 (66.7%)	4 (50.0%)	
PD	0 (0%)	1 (12.5%)	
Missing	1 (11.1%)	0 (0%)	
ICI type			
Anti–PD-1	7 (77.8%)	7 (87.5%)	1.00
Anti–PD-L1	2 (22.2%)	1 (12.5%)	
Number of ICI cycles			
Mean (SD)	3.22 (2.39)	1.63 (0.744)	0.12
Median [min, max]	2.00 [1.00, 8.00]	1.50 [1.00, 3.00]	
Times to diagnosis			
Mean (SD)	78.9 (52.0)	33.8 (22.2)	0.06
Median [min, max]	69.0 [7.00, 155]	34.5 [4.00, 57.0]	

ICI, immune checkpoint inhibitor; Chemo, chemotherapy.

### Laboratory and Other Relevant Examination Results

Baseline laboratory results were available in the majority of our patients. Troponin was elevated in all of the patients. Troponin values in most patients did not return to normal after 6 months, and the levels fluctuated considerably during therapy. The median troponin levels were significantly higher in the severe myocarditis group than in patients with mild myocarditis (61.2 ng/L vs. 440 ng/L, respectively, *p* = 0.0057). B-type natriuretic peptide (BNP), creatine kinase-MB (CK-MB), and myoglobin in the severe cohort were higher than in the mild cohort, but the difference was not statistically significant. A significant elevation in lactate dehydrogenase (LDH) was observed between those two groups (*p* = 0.04). The median value of LDH was 233 IU/L and 519 IU/L in the mild and severe myocarditis groups, respectively. White blood cells (WBC), neutrophils, lymphocytes, eosinophils, and monocytes were higher in the severe group than in the mild group, but those parameters were not statistically significant (Table 3). CD3, CD8, and CD4 serum levels were also examined prior to ICI therapy, but no significant differences were seen. Blood lipid levels, including total cholesterol (TC), triglycerides (TG), low-density lipoprotein cholesterol (LDL-C), and high-density lipoprotein cholesterol (HDL-C), were also measured in our study. No statistical significance was detected between the severity of myocarditis and blood lipid levels. Higher TC and LDL-C levels and lower HDL-C levels were found in the severe group, and large-scale studies considering the relationship are needed for further exploration.

**TABLE 3:** Laboratory result, cardiac markers, and ECG in all patients.

	Overall (*n* = 18)	Mild group (*n* = 12)	Severe group (*n* = 6)	*p*-value
WBC*109/L				
Mean (SD)	5.37 (1.65)	4.94 (1.42)	6.23 (1.87)	0.15
Median [min, max]	4.85 [2.52, 8.79]	4.70 [2.52, 7.77]	5.77 [4.53, 8.79]	
Neutrophils*109/L				
Mean (SD)	3.51 (1.86)	3.07 (1.69)	4.38 (2.03)	0.21
Median [min, max]	3.20 [1.20, 8.10]	2.81 [1.20, 6.13]	3.39 [2.85, 8.10]	
Lymphocytes*109/L				
Mean (SD)	1.19 (0.70)	1.17 (0.69)	1.22 (0.77)	1.00
Median [min, max]	0.91 [0.31, 2.37]	0.90 [0.43, 2.37]	0.99 [0.31, 2.25]	
Eosinophils*109/L				
Mean (SD)	0.15 (0.14)	0.17 (0.16)	0.10 (0.10)	0.37
Median [min, max]	1.00 [0, 0.48]	1.00 [0.02, 0.48]	0.07 [0, 0.23]	
Monocytes*109/L				
Mean (SD)	0.41 (0.15)	0.37 (0.15)	0.49 (0.12)	0.17
Median [min, max]	0.41 [0.06, 0.64]	0.40 [0.06, 0.52]	0.45 [0.36, 0.64]	
LDH (IU/L)				
Mean (SD)	485 (455)	358 (342)	740 (575)	0.04
Median [min, max]	260 [144, 1760]	233 [144, 1,350]	519 [230, 1760]	
CK-MB (ng/ml)				
Mean (SD)	47.8 (76.0)	26.8 (38.2)	89.7 (115)	0.28
Median [min, max]	8.30 [0.89, 300]	7.22 [0.89, 125]	44.5 [1.79, 300]	
Myoglobin (ng/ml)				
Mean (SD)	688 (912)	505 (751)	1,090 (1,160)	0.14
Median [min, max]	246 [32.7, 3,000]	188 [32.7, 2,660]	902 [123, 3,000]	
Missing	2 (11.1%)	1 (8.3%)	1 (16.7%)	
Troponin (ng/L)				
Mean (SD)	359 (554)	134 (195)	810 (772)	0.0057
Median [min, max]	111 [22.5, 1990]	61.2 [22.5, 715]	440 [130, 1990]	
BNP (ng/L)				
Mean (SD)	2,510 (6,980)	774 (1,340)	5,700 (11,600)	0.34
Median [min, max]	312 [19.0, 29,200]	212 [19.0, 4,620]	933 [85.0, 29,200]	
Missing	1 (5.6%)	1 (8.3%)	0 (0%)	
PCT (ng/ml)				
Mean (SD)	1.07 (1.54)	0.78 (0.960)	1.48 (2.18)	0.87
Median [min, max]	0.18 [0.02, 5.02]	0.22 [0.02, 2.12]	0.13 [0.03, 5.02]	
Missing	6 (33.3%)	5 (41.7%)	1 (16.7%)	
IL-6 (pg/ml)				
Mean (SD)	265 (805)	401 (1,010)	26.3 (32.4)	0.92
Median [min, max]	12.8 [2.29, 2,690]	12.8 [2.29, 2,690]	12.6 [2.77, 77.1]	
Missing	7 (38.9%)	5 (41.7%)	2 (33.3%)	
CRP (mg/L)				
Mean (SD)	45.6 (42.4)	53.1 (49.5)	34.9 (32.0)	0.74
Median [min, max]	42.7 [2.97, 131]	68.5 [2.97, 131]	22.2 [4.75, 74.9]	
Missing	6 (33.3%)	5 (41.7%)	1 (16.7%)	
NLR				
Mean (SD)	5.00 (6.05)	4.10 (3.56)	6.81 (9.51)	0.54
Median [min, max]	3.04 [0.51, 26.1]	2.71 [0.51, 10.6]	3.09 [1.59, 26.1]	
CD3				
Mean (SD)	64.3 (16.0)	66.0 (19.2)	62.0 (11.6)	0.76
Median [min, max]	63.6 [39.6, 90.1]	64.7 [39.6, 90.1]	62.4 [46.6, 76.7]	
CD4				
Mean (SD)	28.7 (12.1)	31.7 (14.3)	24.5 (7.6)	0.53
Median [min, max]	27.1 [16.1, 58.3]	29.5 [16.1, 58.3]	22.1 [17.1, 35.8]	
CD8				
Mean (SD)	31.3 (15.2)	30.6 (17.7)	32.2 (12.9)	0.64
Median [min, max]	27.5 [17.0, 68.6]	27.4 [17.0, 68.6]	31.3 [17.7, 49.4]	
Blood lipid levels				
TC (mmol/L)	4.44 (0.876)	4.39 (1.02)	4.53 (0.569)	0.49
TG (mmol/L)	1.73 (0.702)	1.77 (0.744)	1.66 (0.671)	0.82
HDL-C (mmol/L)	1.08 (0.302)	1.19 (0.227)	0.857 (0.328)	0.06
LDL-C (mmol/L)	2.69 (0.783)	2.52 (0.904)	3.02 (0.302)	0.06
ECG				
Abnormal T wave	7/18	4/12	3/6	0.63
Abnormal ST segment	3/18	3/12	0/6	0.52
Conduction defects	3/18	2/12	1/6	1.00
Sinus tachycardia	3/18	2/12	1/6	1.00
Normal ECG	1/18	1/12	0/6	-

CK-MB, creatine kinase-MB; BNP, B-type natriuretic peptide; CRP, C-reactive protein; ECG, electrocardiographic; LDH, lactate dehydrogenase; NLR, neutrophil–lymphocyte ratio; TC, total cholesterol; TG, triglycerides; LDL-C, low-density lipoprotein cholesterol; HDL-C, high-density lipoprotein cholesterol.

Every patient received an electrocardiographic (ECG) examination, and nearly every case of myocarditis showed an irregular ECG (94.4%). It was found that seven cases had abnormal T waves, three cases had an abnormal ST segment, and conduction defects were also found in three patients. In addition, sinus tachycardia showed in three cases. But such a difference in ECG between those two groups was not statistically significant.

Echocardiography was performed on 17 patients. Two patients were discovered to have a ventricular wall movement disorder, and two patients had left ventricular systolic dysfunction (LVEF ≤ 50%). One of those two patients was diagnosed with severe myocarditis and third-degree atrioventricular block, with an LVEF of 40%. Coronary angiography was also performed on this patient, which revealed the anterior descending branch, circumflex branch, and right coronary artery were stenosed, and the first diagonal branch had the most severe stenosis of nearly 40%. However, the patient had no history of coronary heart disease. Coronary angiography results were also obtained from the other four patients. One patient in the severe group had stenosis in the left anterior descending branch, circumflex branch, and right coronary artery. The diagnosis of myocarditis in these two cases was made by a multidisciplinary team, and they all matched the inclusion criteria. Nevertheless, there were no meaningful findings in the other three patients in the mild group. A cardiac magnetic resonance imaging (MRI) investigation was performed in six cases; however, we were unable to find meaningful data in five of them. Only one patient showed myocardial edema on T2-weighted imaging. Furthermore, one patient with severe myocarditis received cardiac 18F-fluorodeoxyglucose (18F-FDG) position emission tomography magnetic resonance imaging (PET-MRI), which revealed enhanced FDG metabolism at the left ventricle. Once myocarditis was diagnosed, patients received steroid treatment. The initial steroid dose was an equivalent of methylprednisolone (1–2 mg/kg), which was sustained for 3–5 days and followed by a long-term oral steroid taper. In four patients, intravenous gamma globulin was used to relieve their symptoms. Unfortunately, one patient died as a result of myocarditis-related complications.

### Concomitant irAEs and Outcomes

**FIGURE 2:**
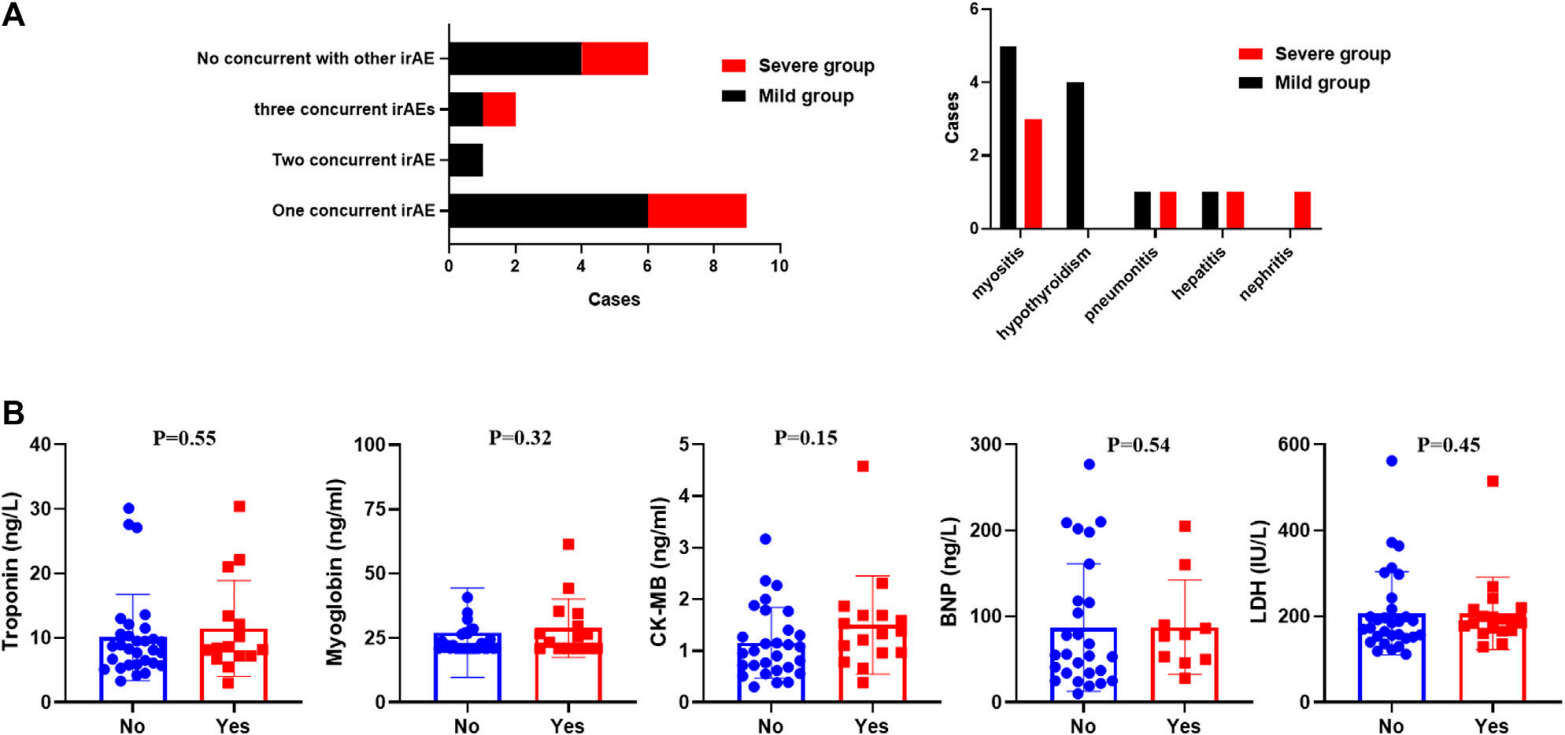
**(A)** Distribution of concurrent irAEs in our study. **(B)** Serum myocardial enzymes and LDH between patients with or without immune-related myocarditis.

66.7% of patients (12 cases) experienced at least one irAE (Figure 2A). A total of nine patients developed another irAE: six patients were diagnosed with immune-related myositis, two acquired hypothyroidism, and one suffered pneumonitis. One patient developed another two irAEs, and two patients were concurrent with three irAEs. Overall, there was no difference in the prevalence between the mild and severe groups (*p* > 0.5). Myositis (*n* = 8) and hypothyroidism (*n* = 4) were the most common irAEs in our cohorts.

The difference between those who had additional irAE and those who did not was also discussed in our research (Table 4). Patients without concomitant irAEs (*n* = 6) were all males and treated with PD-1 antibodies. Patients with a single irAE had a delayed onset time than those with multiple irAEs. Baseline characteristics were explored, and we identified that the alcohol history was significantly different. All patients with concomitant irAEs did not have an alcohol history (*p* = 0.03). However, this result should be validated in a larger cohort study. There were also statistically significant changes in LDH and myoglobin. Patients with multi-irAEs had a higher levels of LDH (250 IU/L vs. 603 IU/L; *p* = 0.02) and myoglobin (112 ng/ml vs. 950 ng/ml; *p* = 0.02). There was also a trend of higher CK-MB, BNP, and a lower troponin in patients with multi-irAEs, although these changes were not statistically significant.

**TABLE 4:** Comparison of myocarditis patients with single irAE or multi-irAE.

	Single irAE (*n* = 6)	Multi-irAE (*n* = 12)	*p-*value
Severity			
Mild	4 (66.7%)	8 (66.7%)	1
Severe	2 (33.3%)	4 (33.3%)	
Age	65.7 (10.5)	53.8 (16.0)	0.12
Gender			
Male	6 (100%)	9 (75.0%)	0.52
Female	0 (0.00%)	3 (25.0%)	
Cancer type			
Other cancers	2 (33.3%)	2 (16.7%)	0.42
Lung cancer	4 (66.7%)	6 (50.0%)	
Thymic carcinoma	0 (0.00%)	4 (33.3%)	
ICI type			
Anti-PD-1	6 (100%)	9 (75.0%)	0.52
Anti-PD-L1	0 (0.00%)	3 (25.0%)	
Number of ICI cycles	3.33 (2.50)	1.92 (1.44)	0.12
Times to onset	72.0 (50.7)	46.8 (42.8)	0.28
WBC*10^9^/L	4.86 (0.99)	5.63 (1.89)	0.28
Neutrophils*10^9^/L	3.26 (0.70)	3.64 (2.25)	0.74
Lymphocytes*10^9^/L	1.07 (0.65)	1.24 (0.74)	0.85
Monocytes*10^9^/L	0.40 (0.26)	0.42 (0.06)	0.48
Eosinophils*10^9^/L	0.11 (0.09)	0.17 (0.16)	0.74
NLR	4.25 (3.00)	5.38 (7.21)	0.67
CK-MB (ng/ml)	8.60 (10.4)	67.3 (87.3)	0.08
Myoglobin (ng/ml)	112 (65.0)	950 (1,003)	0.02
Troponin (ng/L)	479 (763)	299 (445)	0.89
BNP (ng/L)	992 (1,042)	3,341 (8,679)	0.72
PCT (ng/ml)	2.45 (2.42)	0.61 (0.91)	0.12
CRP (mg/L)	30.6 (42.1)	50.5 (43.7)	0.64
LDH (IU/L)	250 (107)	603 (519)	0.02
IL-6 (pg/ml)	1,351 (1893)	23.2 (30.1)	0.34
CD3	65.3 (17.6)	64.0 (16.5)	1.00
CD4	40.0 (16.6)	24.9 (8.26)	0.15
CD8	22.2 (7.91)	34.3 (16.2)	0.21

*Data are shown as means ± SD.

CK-MB, creatine kinase-MB; BNP, B-type natriuretic peptide; CRP, C-reactive protein; ECG, electrocardiographic; LDH, lactate dehydrogenase; NLR, neutrophil–lymphocyte ratio.

### Comparison of the Baseline Levels of Serum Myocardial Enzymes and Lactate Dehydrogenase Between Different Groups

Our results revealed that the serum myocardial enzymes and LDH might play a suggestive role in the early diagnosis of immune-related myocardial injury. However, baseline values of those markers did not differ statistically between patients with severe and mild myocarditis in our study (*p* > 0.05, Table 5). To further explore the difference in those markers between patients with and without myocarditis, we retrospectively screened 30 patients without myocarditis in our hospital who were treated with ICIs. There was no statistical significance in the differences in serum myocardial enzymes and LDH between patients who experienced immune-related myocarditis and those who did not experience them (*p* > 0.05, Figure 2B). We also analyzed the changes in those markers during treatment with ICIs in patients without immune-associated myocarditis. The comparison of LDH, troponin, CK-MB, BNP, and myoglobin levels between baseline and after one or two ICI cycles were not statistically significant (*p* > 0.05).

**TABLE 5:** Comparison of the baseline levels of serum myocardial enzymes and LDH between different groups.

	Mild group	Severe group	*p*-value
Troponin (ng/L)	12.9 (8.52)	8.58 (3.79)	0.67
Myoglobin (ng/ml)	31.3 (13.2)	24.1 (3.61)	0.71
CK-MB (ng/ml)	1.49 (1.12)	1.54 (0.546)	0.57
BNP (ng/L)	94.5 (57.5)	59.0 (43.8)	0.67
LDH (IU/L)	221 (99.5)	179 (29.2)	0.34

### Role of Troponin and Lactate Dehydrogenase Levels in Diagnosing Severe Myocarditis

We searched PubMed for relevant articles to validate our findings and selected 30 patients for analysis (Katsume et al., 2018; Khoury et al., 2019; Wang and Hu, 2019; Balanescu et al., 2020; Diamantopoulos et al., 2020; Hardy et al., 2020; Hyun et al., 2020; Leaver et al., 2020; Szuchan et al., 2020; Tu et al., 2020; Valenti-Azcarate et al., 2020; Wang et al., 2020; Xie et al., 2020; Xing et al., 2020; Arangalage et al., 2021; Cham et al., 2021; Chen et al., 2021; Elder et al., 2021; Iwasaki et al., 2021; Komatsu et al., 2021; Liang et al., 2021; Tanabe et al., 2021; Yanase et al., 2021; Zhang et al., 2021). The results are coincident with ours. Patients with severe myocarditis have a higher troponin (*p* = 0.005) and an earlier onset time (19.1 vs. 29.7 days, *p* = 0.014) than patients in the mild group. In addition, a significant difference was found in the outcome between the two groups (Table 6). Patients in the severe group have a higher mortality (53.3% vs. 6.7%, *p* = 0.041). The LDH in most studies were not eligible and, thus, we did not analyze it.

**TABLE 6:** Patients with immune-related myocarditis searched in PubMed were analyzed.

	Mild group (*n* = 15)	Severe group (*n* = 15)	*p-*value
Number of ICI cycles			
Mean (SD)	1.53 (1.06)	1.27 (0.458)	0.64
Median [min, max]	1.00 [1.00, 5.00]	1.00 [1.00, 2.00]	
Times to diagnosis			
Mean (SD)	29.7 (20.8)	19.1 (9.38)	0.041
Median [min, max]	28.0 [4.00, 90.0]	19.0 [4.00, 45.0]	
Troponin (ng/L)			
Mean (SD)	1,200 (1,280)	4,540 (4,580)	0.005
Median [min, max]	1,030 [44.0, 3,720]	3,230 [806, 16,300]	
Outcomes			
Lived	14 (93.3%)	7 (46.7%)	0.014
Died	1 (6.7%)	8 (53.3%)	

## Discussion

Immune checkpoint inhibitors have drastically improved clinical outcomes, and they are increasingly being licensed for use in the early stage of cancer and in combination with other anti-tumor therapies, such as chemotherapy or targeted therapy (Postow et al., 2015; Zhang et al., 2018; Luo et al., 2021). However, due to the increased usage of these medications, immune-related adverse events in patients receiving ICIs are becoming more widely recognized, which may limit their clinical applicability. One of the most fatal irAEs is immune-related myocarditis. Due to its severity and high mortality, there is a growing interest in further study (Ganatra and Neilan, 2018). Because of its rarity, the diagnosis and management of ICI-related myocarditis represent a clinical challenge. The diagnostic algorithm may start with troponin screening or the onset of symptoms during ICI therapy (Ball et al., 2019; Hu et al., 2019).

The risk of myocarditis varies depending on the treatment regimen and the ICI drugs used. Overall, ICI-related myocarditis was reported to be around 1% of the incidence. Previous research showed that dual ICIs regimens caused a higher incidence than monotherapy (Rubio-Infante et al., 2021). The incidence of monotherapy, dual ICIs therapies, and ICI plus chemotherapy were found to be 3.1, 5.8, and 3.7%, respectively. There were also variations in the occurrence of myocarditis among the various classes of ICIs (Mahmood et al., 2018). Based on results from limited clinical studies, myocarditis is more likely to be caused by the CTLA-4 antibody (Mascolo et al., 2021). Furthermore, it has been observed that nivolumab (anti-PD-1) had a decreased incidence of cardiac irAEs. The underlying mechanism is still unclear. Many hypotheses have been formulated with regard to the relationship between ICIs and cardiotoxicity. T-cell infiltration into the myocardium, increased auto-antibodies acting on self-antigens, and increased T cells reacting to antigens shared by cancer and normal cells could all be contributors to the pathomechanism of the irAEs (Baik et al., 2021).

There is a scarcity of information on this potentially fatal adverse event. Our current understanding of the illness is limited, and patient-derived evidence is scarce. Recent research has concentrated on examining the disease’s overall characteristics while neglecting the individual variabilities. The goal of this retrospective study was to provide new insights about such a rare irAE and explore risk factors for severe myocarditis and multi-irAEs. According to Common Terminology Criteria for Adverse Events (CTCAE), AE grading is currently based on the outcomes of biomarkers, ECG, symptoms, and cardiac complications (Brahmer et al., 2018). Patients with grade 1 need to be closely monitored during therapy. The patient in grade 1 was asymptomatic but had aberrant cardiac biomarkers with an irregular ECG. Compared to grade 1, patients with grade 2 had minor symptoms. Symptoms in grade 3 patients were more severe and required the use of steroids to manage. Grade 4 was defined as moderate to severe decompensation of life-threatening situations that demand intravenous injection of medication or intervention. Patients were divided into two groups in our study based on their clinical manifestations and whether or not they had hemodynamic complications such as heart failure, cardiogenic shock, or arrhythmia, which may be more useful in assisting clinicians in determining a patient’s condition and prognosis. Patients in the severe group were categorized as grade 3–4 toxicity, and patients in the mild group were classified as grade 1–2 toxicity.

It is critical to distinguish between mild and severe myocarditis due to the high mortality rate. Several studies have identified treatment regimens, comorbidities such as hypertension and diabetes, and tobacco use as risk factors for cardiotoxicity (Patel et al., 2021). However, Subgroup analyses based on age, gender, tobacco usage, diabetes, and cancer stage revealed no differences in our study. According to our findings, hypertension and cancer type may not correlate to the severity of myocarditis. Contrary to earlier reports, our data have shown that none of the patients in the severe group had hypertension. In addition, none of the 18 patients had coronary artery disease or any other type of cardiac disease. No statistical significance was detected between the severity of myocarditis and blood lipid levels.

The most common malignancy type in the mild group was lung cancer, while the most common type in the severe group was thymic carcinoma. The fundamental cause of the discrepancy in our study was unknown. This could be due to the fact that a large percentage of patients with thymic cancer have an autoimmune syndrome, which could be an independent risk factor for ICI-associated cardiotoxicity. The thymus plays an essential role in the development of T-cells. Although autoimmune disease patients are not the focus of our research, the function and composition of T-cells, which are important components of the immune system, are similarly aberrant in the thymoma microenvironment (Hung et al., 2019). In addition, the incidence of myocarditis was higher in two trials which evaluated pembrolizumab in thymic tumors, at 5 and 9.1%, respectively (Giaccone et al., 2018; Cho et al., 2019). Additional studies are needed to further explore the link between myocarditis, thymic tumors, and ICI therapy. Overall, monitoring the occurrence of myocarditis in thymic carcinoma patients receiving ICI is crucial.

According to the literature, immune-related myocarditis frequently develops soon after starting ICI therapy. The median time of onset was 34 days, with the majority of cases occurring within 3 months (Mahmood et al., 2018). In our studies, the median length was 2 cycles, and the onset time was 51 days (range: 4–155), which is longer than previous reports (Ma et al., 2021). Patients with severe myocarditis had a faster onset time than those with mild myocarditis. Myocarditis is more likely to present at an early age in patients in the mild group who were treated with combination therapy, although the results of onset time were not statistically significant. Treatment lines, ICI type, number of ICI cycles, and ICI efficacy all had no statistically significant differences. Recent research suggested that irAEs have been linked to a long-term response and therapeutic benefit (Dimitriou et al., 2021; Ma et al., 2021; Paderi et al., 2021). Among the 16 patients, five patients had a partial response (PR), ten patients had stable disease (SD), and one patient had progressive disease (PD).

Early identification of severe myocarditis followed by timely treatment is essential to reduce mortality and assist physicians in personalized medicine decision-making. Our findings showed that troponin and LDH might contribute to recognizing severe myocarditis as soon as possible. Previous studies have demonstrated that monitoring troponin during treatment is reasonable (Sarocchi et al., 2018; Liu et al., 2021; Puzanov et al., 2021), but the relationship between LDH and cardiotoxicity was never reported. Patients with severe myocarditis are more likely to have a higher LDH. Furthermore, real-time screening of concomitant irAE is essential in patients experiencing immune-related myocarditis, especially in patients with higher LDH and myoglobin levels. In addition, the level of troponin also correlated with outcomes.

The diagnosis and management of this disorder continue to be a clinical and research challenge. Myocardial biopsy is the gold standard for diagnosis. However, the complications may have an adverse effect on clinical utility and outcomes (Imran et al., 2019). The relevance of cardiac MRI, FDG-PET, and coronary angiography in the identification of myocarditis has also been highlighted in recent research (Nensa et al., 2018; Spallarossa et al., 2020; Ederhy et al., 2021). However, performing those examinations may miss the optimal treatment time and result in negative repercussions. A multidisciplinary team (MDT) approach is crucial for assessing suspected immune-related myocarditis since it aids decision-making and lowers death rates. The management of ICI-related myocarditis demands close collaboration between oncologists and cardiologists.

The mortality rate was reported to be as high as 46% (Mahmood et al., 2018), while in our study, it was 5.6% (1/18). Early identification by MDT and the use of steroids may have contributed to the lower mortality rate. For severe myocarditis, there is no question that corticosteroids should be used, but the treatment of mild myocarditis by steroids is still unknown and requires further research. Our study provides clinical relevance and rationale for initiating corticosteroid therapy regardless of the severity of myocarditis. All 18 patients in our study received steroid treatment. An equivalent dose of (methyl) prednisolone (1–2 mg/kg) was given for the first 3–5 days, followed by a long-term oral steroid taper. In addition to the use of steroids, intravenous gamma globulin should be considered to alleviate the symptoms of patients based on their clinical needs. After being diagnosed with myocarditis, no one disputes ICIs again.

**FIGURE 3:**
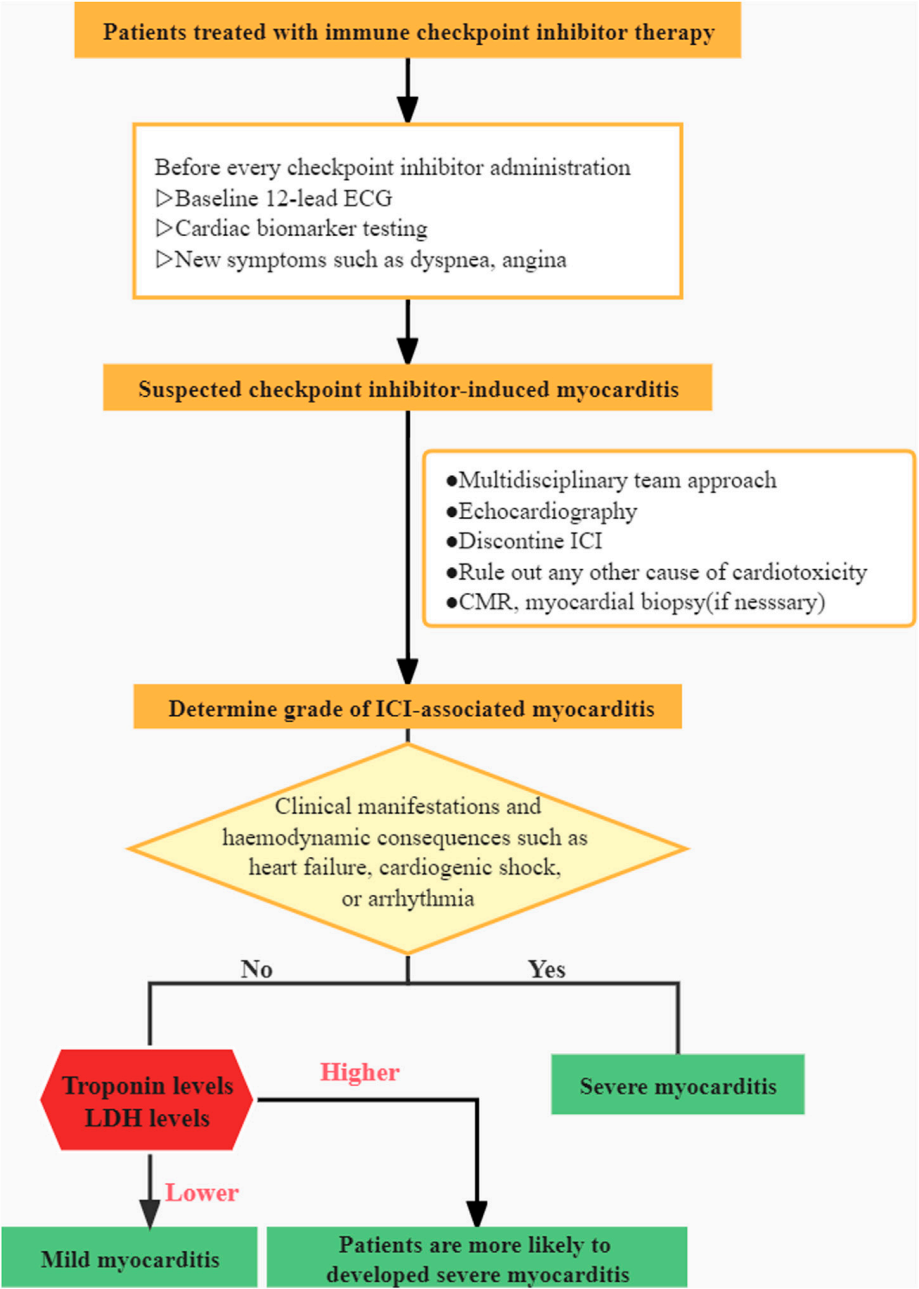
Algorithm for the management of immune-related myocarditis.

Our research fills a gap in the literature and sheds new light on a rare irAE. A clinical algorithm is presented based on our clinical experience (Figure 3). However, there are a few flaws worth mentioning. It is worth noting the inherent bias in any single-institution retrospective analysis and also the limited sample population in our study. Due to the challenges in diagnosis, the vast majority of myocarditis events may be overlooked or misdiagnosed. Furthermore, extreme vigilance is essential in the therapy and the avoidance of excessive corticosteroid usage for biased results. Further investigation is required as an outcome of our findings.

## Data Availability

The raw data supporting the conclusions of this article will be made available by the authors, without undue reservation.
